# Responses of five Mediterranean halophytes to seasonal changes in environmental conditions

**DOI:** 10.1093/aobpla/plu049

**Published:** 2014-08-19

**Authors:** Ricardo Gil, Inmaculada Bautista, Monica Boscaiu, Antonio Lidón, Shantanu Wankhade, Héctor Sánchez, Josep Llinares, Oscar Vicente

**Affiliations:** 1 Instituto de Biología Molecular y Celular de Plantas (UPV-CSIC), Universitat Politècnica de València, Camino de Vera s/n, 46022 Valencia, Spain; 2 ReForest, Departamento de Ingeniería Hidráulica y Medio Ambiente, Universitat Politècnica de València, Camino de Vera s/n, 46022 Valencia, Spain; 3 Instituto Agroforestal Mediterráneo (IAM), Universitat Politècnica de València, Camino de Vera s/n, 46022 Valencia, Spain; 4 Instituto de Investigación para la Gestión Integral de Zonas Costeras (IGIC), Universitat Politècnica de València, C/Paranimf 1, 46730 Grao de Gandia, Valencia, Spain

**Keywords:** Drought, *Inula crithmoides*, *Juncus acutus*, *Juncus maritimus*, littoral salt marsh, Mediterranean climate, oxidative stress, *Plantago crassifolia*, *Sarcocornia fruticosa*, soil salinity.

## Abstract

In their natural habitats, different mechanisms may contribute to the tolerance of halophytes to high soil salinity and other abiotic stresses, but their relative contribution and ecological relevance, for a given species, remain largely unknown. We studied the responses to changing environmental conditions of five halophytes (*Sarcocornia fruticosa*, *Inula crithmoides*, *Plantago crassifolia*, *Juncus maritimus* and *J. acutus*) in a Mediterranean salt marsh, from summer 2009 to autumn 2010. A principal component analysis was used to correlate soil and climatic data with changes in the plants' contents of chemical markers associated with stress responses: ions, osmolytes, malondialdehyde (MDA, a marker of oxidative stress) and antioxidant systems. Stress tolerance in *S. fruticosa*, *I. crithmoides* and *P. crassifolia* (all succulent dicots) seemed to depend mostly on the transport of ions to aerial parts and the biosynthesis of specific osmolytes, whereas both *Juncus* species (monocots) were able to avoid accumulation of toxic ions, maintaining relatively high K^+^/Na^+^ ratios. For the most salt-tolerant taxa (*S. fruticosa* and *I. crithmoides*), seasonal variations of Na^+^, Cl^−^, K^+^ and glycine betaine, their major osmolyte, did not correlate with environmental parameters associated with salt or water stress, suggesting that their tolerance mechanisms are constitutive and relatively independent of external conditions, although they could be mediated by changes in the subcellular compartmentalization of ions and compatible osmolytes. Proline levels were too low in all the species to possibly have any effect on osmotic adjustment. However—except for *P. crassifolia*—proline may play a role in stress tolerance based on its ‘osmoprotectant’ functions. No correlation was observed between the degree of environmental stress and the levels of MDA or enzymatic and non-enzymatic antioxidants, indicating that the investigated halophytes are not subjected to oxidative stress under natural conditions and do not, therefore, need to activate antioxidant defence mechanisms.

## Abstract (precis)

We have studied the responses to changing environmental conditions of five halophytes in a Mediterranean salt marsh, during a 2-year period. Salt tolerance in succulent dicotyledonous halophytes is mostly dependent on compartmentalisation of toxic ions in vacuoles and biosynthesis of osmolytes for osmotic adjustment – mechanisms that appear to be constitutive in the most tolerant taxa – while monocots avoid excessive ion transport to the plant aerial parts. Contrary to what has been described for salt treatments under artificial conditions, the selected halophytes are not affected by oxidative stress in their natural habitat, and do not need to activate antioxidant defence mechanisms.

## Introduction

Soil salinity is, together with drought, one of the most important environmental conditions that reduce crop yields worldwide and determine the distribution of wild plants in nature (Boyer 1982; Bartels and Sunkar 2005; Watson and Byrne 2009). Studies of plant responses to salt—as well as to other abiotic stress factors—and the elucidation of stress tolerance mechanisms have become one of the most active areas of research in plant biology due to their academic and practical interest. There is now substantial evidence that all plants react against adverse environmental conditions by activating a series of conserved responses which are common to different abiotic stresses. One of these basic stress responses involves ion homoeostasis and the maintenance of osmotic balance to counteract cellular dehydration caused by, for example, high soil salinity, drought, cold or high temperatures: limitation of water losses, sequestration of toxic ions in the vacuole (according to the so-called *ion compartmentalization hypothesis*; Flowers *et al.* 1977; Wyn Jones *et al.* 1977; Glenn *et al.* 1999) and synthesis and accumulation of compatible solutes or osmolytes in the cytoplasm (Munns and Termaat 1986; Zhu 2001; Munns and Tester 2008; Kronzucker and Britto 2011). These latter compounds, apart from contributing to osmotic adjustment, play ‘osmoprotectant’ roles by acting as low-molecular-weight chaperones to stabilize proteins, membranes and other macromolecular structures under stressful conditions, and also as scavengers of ‘reactive oxygen species’ (ROS) (Ashraf and Foolad 2007; Chen and Murata 2008; Flowers and Colmer 2008; Hussain *et al.* 2008; Szabados and Savoure 2010). Most stressful environmental factors cause oxidative stress in plants through generation of ROS; consequently, another fundamental and conserved response to abiotic stress consists of the activation of enzymatic and non-enzymatic antioxidant systems to avoid or reduce oxidative damage of proteins, membranes and DNA (Apel and Hirt 2004; Halliwell 2006; Miller *et al.* 2008; Türkan and Demiral 2009). Paradoxically, the vast majority of studies on salt stress responses and salt tolerance mechanisms have been performed using glycophytes (salt-sensitive plants), many with the model species *Arabidopsis thaliana* (e.g. Zhu 2000, 2002; Koiwa *et al.* 2006; Horie *et al.* 2009; and references therein). Nevertheless, there is a growing interest in the study of salt-tolerant plants—halophytes—which *a priori* seem to be more appropriate models to elucidate these mechanisms. Research on halophytes' responses to salt stress has indeed increased in recent years and has provided information on the molecular, biochemical and physiological bases of their tolerance to high soil salinity, which appear to rely on the activation of the aforementioned general stress responses also used by salt-sensitive species, albeit with much lower efficiency. Yet important aspects of these mechanisms remain largely unknown, especially regarding the ecological relevance of distinct response mechanisms and their relative contribution to salt tolerance in particular halophytic taxa. Most studies on halophytes' behaviour under high salinity conditions have been conducted in artificial laboratory or greenhouse environments, with the obvious advantage of allowing strict experimental control, but which cannot reflect the real conditions of plants in nature. In their natural habitats, halophytes must react dynamically to changing, uncontrollable environmental conditions. Halophytes must cope simultaneously with different abiotic stresses, not only soil salinity, which may activate the same, overlapping and/or specific responses, interacting in complex ways rather than showing simple additive effects (Ungar 1991; Krasensky and Jonak 2012; Ben Hamed *et al.* 2013). For these reasons, the interpretation of data obtained in the field is much more difficult than the analysis of laboratory results. It is, therefore, not surprising that very few studies have been published dealing with the stress responses of halophytes under varying environmental conditions in their natural ecosystems (e.g. Doddema *et al.* 1986; Murakeözy *et al.* 2002, 2003; Walker *et al.* 2008; Mouri *et al.* 2012; Boscaiu *et al.* 2013).

Despite the potential difficulties, we have recently proposed that research on the dynamic behaviour of halophytes in their natural habitats should be intensified as a complementary approach to laboratory experiments (Gil *et al.* 2013). This will likely provide novel information, allowing us to deepen our understanding of the stress tolerance mechanisms of halophytes—and of plants in general—but in natural, ecologically relevant circumstances. Our strategy in these studies is based on the analysis of changes in the levels of biochemical markers associated with particular stress responses in the samples collected from plants growing in natural habitats under different environmental conditions. We assume that, for a given species, a significant correlation between these changes and the variations in the type and intensity of abiotic stress affecting the plants would provide evidence for the relevant participation of the specific stress response in its physiological mechanisms of stress tolerance.

For the work described herein, and following the strategy outlined in the previous paragraph, we selected five perennial halophytes, three succulent dicotyledonous species (*Sarcocornia fruticosa*, *Inula crithmoides* and *Plantago crassifolia*) and two related monocotyledonous taxa (*Juncus maritimus* and *J. acutus*), to analyse their responses to environmental stress in their natural habitat, a littoral salt marsh in the province of Valencia (East Spain), over an almost 2-year period. The specific aim of the work was to check whether statistically significant correlations could be established between particular stress response mechanisms (ion uptake, accumulation of specific osmolytes, activation of antioxidant systems) and soil parameters and climatic data associated with environmental stress. In this way, we expected to establish which responses are relevant for stress tolerance under natural conditions, and which are not, for each species investigated.

Some results of this project, specifically the determination of soluble carbohydrate (sugars and polyols) contents in the same plant samples, have been previously published (Gil *et al.* 2011). Those data are not shown herein, but have been included, together with the present results, in the analysis of statistical correlations with environmental parameters by principal component analysis (PCA).

## Methods

### Study site

The study was carried out in a littoral salt marsh 15 km south of the city of Valencia (East Spain), in ‘La Albufera’ Natural Park (39°47′28″N, 1°04′25″W). La Albufera is the biggest lake in the Iberian Peninsula originating from an ancient marine gulf that was gradually closed by littoral sandy strips. The salt marsh is located in an inter-dune depression between the first belt of dunes close to the sea and older stabilized dunes closer to the fresh-water lake. The climate there is of the thermomediterranean thermotype. Due to its coastal location, there is little temperature variation. The yearly mean temperature is ∼17.5 °C; the warmest month of the year is August, with means of ∼25 °C, and the coldest month is January, with means of 10 °C. The ombrotype is dry, with mean annual rainfall at around 450 mm, but with broad variations in different years [ombrotypes, which represent ombroclimatic belts, are based on the ombrothermic index (*I*_o_) which is calculated as a function of both the total positive precipitation and temperature]. The wettest season is generally autumn, especially October, followed by spring. As is typical of Mediterranean climate, summers are dry (Rivas-Martínez and Rivas-Saenz 1996–2009) with a negative water balance.

### Selected halophytic taxa

The five selected halophytes were perennial species, to allow plant material collection from the same individuals throughout the study period.

*Sarcocornia fruticosa* belongs to the family Amaranthaceae, whose stem-succulent genera are considered among the most salt-tolerant halophytes (Short and Colmer 1999). It is a small shrub of up to ≥1 m in height, erect and much branched, with fleshy-articulated stems, woody only in the basal part and with scale-like leaves. It seems to have adapted to a wide range of salinities including extremely hypersaline conditions, indicating considerable physiological plasticity (Redondo-Gómez *et al.* 2006). The species is frequent in plant communities of the vegetation class *Arthrocnemetea* growing mainly in strongly saline, more or less moist soils, which may occasionally be flooded or inundated by brackish water of marine origin, but may also occur in inner endorheic areas (areas with closed drainage).

*Inula crithmoides* (family Asteraceae) is a small shrub, of up to 1 m high, with linear succulent leaves. It is frequent in littoral salt marshes, sea beaches, brackish riverbeds and coastal cliffs in the Mediterranean region and the Atlantic coast up to the British Isles. It appears in communities that grow on salty soils, with a silty texture and temporarily waterlogged, mostly in littoral areas since it requires mild temperatures. It is common in plant communities of vegetation classes *Arthrocnemetea* and *Juncetea maritimi*.

*Plantago crassifolia* (Plantaginaceae) is a common species on littoral areas in the Mediterranean region and on saline steppes developed on sandy soils. Plants have fleshy, linear rosette leaves and are not as salt-tolerant as the two previous taxa. In the sampling area, this species is abundant in the plant community *Schoeno-Plantaginetum crassifoliae* (vegetation class *J. maritimi*), typical of ecotones, between salt marshes and dune vegetation.

*Juncus maritimus* and *J. acutus* (Juncaceae) are two common rush species, closely related taxonomically, and present on temporally flooded moist soils with a large amount of alkaline carbonates (Fernández-Carvajal 1982). *Juncus acutus* is more competitive on sandy soils with low and moderate salinity, or even gypsicolous (Boira 1995), since it tolerates summer drought conditions well. *Juncus maritimus* requires greater soil moisture, but seems to be more tolerant to salinity, as shown in field and laboratory experiments (Boscaiu *et al.* 2013). In the study area, the two species appear in plant communities of the vegetation class *J. maritimi*.

### Plant material sampling

The fieldwork was carried out over a period of almost 2 years, in 2009 and 2010. An experimental plot of 100 m^2^ (10 m × 10 m), in which all five selected species were present, was established near the flooded area of the salt marsh. Plant material was collected from the same individuals (which were georeferenced and marked in the field) in five successive samplings in summer and autumn 2009 and in spring, summer and autumn 2010 (1 July 2009, 30 November 2009, 19 April 2010, 13 July 2010 and 23 November 2010). Young succulent stems of *S. fruticosa*, young shoots of *I. crithmoides*, leaves of *P. crassifolia* and culms of the two rush species were sampled separately from five individuals per taxon, cooled on ice and transported to the laboratory, where leaves were separated from branches whenever necessary. Part of the plant material was frozen and stored at −75 °C, and the rest was dried in an oven at 65 °C for 3–4 days until constant weight to obtain the percentage of dry weight (DW) of each individual.

### Climatic analysis

To assess the climatic conditions prior to each sampling, weekly data on the mean, maximum and minimum temperatures, rainfall and evapotranspiration (ETP)—recorded by the nearest agroclimatological station, located in Picassent (Valencia), ∼10 km from the experimental area—were obtained from the Agroclimatic Information System for Irrigation of the Spanish Ministry of Environment, Rural and Marine Affairs. The mean temperature and the cumulative values for precipitation and ETP were calculated from the data recorded over a 60-day period prior to each sampling date.

### Soil sampling and analysis

Three representative soil samples were taken from a depth of 0–15 cm simultaneously with each sampling of plant material, in specific zones of the experimental area defined after an intensive analysis of soil salinity carried out before the first sampling in summer 2009. Soil samples to be analysed in the laboratory were air-dried and passed through a 2-mm sieve to remove coarse fragments; previously, a small fraction of each sample was removed to determine soil moisture by weight loss at 105 °C. Soil pH was measured in soil suspensions in a soil-to-water ratio of 1 : 2.5 (w/v) using a Crison MicropH 2001 pH-meter. Electrical conductivity (EC) and ion concentrations were measured in aqueous extracts at a soil-to-water ratio of 1 : 1 (w/v). As reported in the literature, different soil-to-water ratios are used to prepare soil extracts for EC determinations, but soil salinity is most generally defined as the EC measured in so-called saturated soil-pastes (EC_e_, Ayers and Westcot 1985). However, this is not recommended in the case of coarse-textured soils—such as that in the experimental plot—because samples of this type of soil are easily overwetted and small amounts of free water can lead to appreciable errors in the measurements (Rhoades 1996). To avoid this problem without excessive dilution of the samples, we decide to use instead EC_1:1_ values measured in 1 : 1 soil extracts. In addition, preparation of saturated extracts for EC_e_ determinations not only is more complicated to perform but also requires much larger quantities of soil than 1 : 1 extracts, ∼400 g of air-dried soil per sample; with the intensive sampling scheme of our work, this would have seriously affected the experimental plot, and would never be allowed in a protected area such as ‘La Albufera’ Natural Park.

Electrical conductivity was analysed using a Crison 522 conductivity meter, sodium (Na^+^) and potassium (K^+^) with a PFP7 flame photometer (Jenway, Inc., Burlington, USA), chlorides (Cl^−^) with a MKII Chloride Analyzer 926 (Sherwood, Inc., Cambridge, UK) and calcium (Ca^2+^) and magnesium (Mg^2+^) in an atomic absorption spectrometer SpectrAA 220 (Varian, Inc., CA, USA).

### Ion content determination in plant material

Ion measurements were performed according to Weimberg (1987) in aqueous extracts obtained by heating the samples (0.15 g of dried, ground plant material in 10 mL of water) in a water bath, for 1 h at 95 °C, followed by filtration through filter paper (particle retention 8–12 µm). Ions were determined as indicated above for soil analyses: monovalent cations by flame photometry, divalent cations by atomic absorption spectrometry and Cl^−^ with a chloride analyser.

### Osmolyte quantification

Proline (Pro) was extracted with 3 % (w/v) sulfosalicylic acid from 0.1 g of frozen plant material in liquid nitrogen and was quantified according to the acid-ninhydrin method of Bates *et al.* (1973) with minor modifications, as described by Vicente *et al.* (2004).

Glycine betaine (GB) was determined from 0.1 g dry material according to Grieve and Grattan (1983) with the modifications proposed by Nawaz and Ashraf (2010).

The osmolyte concentrations in the plant samples were expressed in µmol g^−1^ of DW.

### Oxidative stress assessment and antioxidant systems

#### MDA determination

Dry plant material (∼100 mg) was ground to a fine powder in a mortar and extracted with 80 % methanol. Samples were shaken gently overnight at room temperature. Supernatants were collected by centrifugation and stored at −20 °C until used in the assays. Malondialdehyde (MDA) was quantified in the extracts by the trichloroacetic/thiobarbituric acid method as described by Hodges *et al.* (1999).

#### Total phenolic compounds and flavonoid assays

Methanolic extracts were prepared as described above for MDA determination, and total phenolic compounds were assayed by reaction with the Folin–Ciocalteu reagent (Singleton and Rossi 1965) using gallic acid as a standard. Phenolic concentrations in the plant samples were expressed as ‘mg equivalent of gallic acid per g of dry weight’. Total flavonoids were determined in the same extracts by a reaction with AlCl_3_ at a basic pH, as described by Zhishen *et al.* (1999), with catechin used as a standard. The plant flavonoid level in each sample was expressed as ‘mg equivalent of catechin per g of dry weight’.

#### Antioxidant enzyme activities

Superoxide dismutase (SOD)-, catalase (CAT)- and glutathione reductase (GR)-specific activities were determined in crude protein extracts prepared from ∼2 g of plant material stored and frozen at −75 °C. Each sample was ground in a mortar in the presence of liquid N_2_ and proteins were extracted with 10–20 mL of extraction buffer [20 mM Hepes, pH 7.5, 50 mM KCl, 1 mM EDTA, 0.1 % (v/v) Triton X-100, 0.2 % (w/v) polyvinylpyrrolidone, 0.2 % (w/v) polyvinylpolypyrrolidone and 5 % (v/v) glycerol], followed by addition of a 1/10 volume of ‘high salt buffer’ (225 mM Hepes, pH 7.5, 1.5 M KCl and 22.5 mM MgCl_2_). Homogenates were centrifuged at 20 000 *g* for 20 min at 4 °C, and the supernatants were concentrated in U-Tube™ concentrators (Novagen, Madison, USA). After removing precipitated material by centrifugation, the final samples (referred to as ‘protein extracts’) were divided into aliquots, frozen in liquid N_2_ and stored at −75 °C until used for enzyme assays. The protein concentration in the extracts was determined by the method of Bradford (1976), using the Bio-Rad reagent and bovine serum albumin as a standard.

##### Superoxide dismutase

Total SOD activity was determined according to Beyer and Fridovich (1987) by monitoring spectrophotometrically the inhibition of nitroblue tetrazolium (NBT) photoreduction. The reaction mixtures (1 mL) contained 50 mM potassium phosphate buffer, pH 7.8, 9.9 mM l-methionine, 58 µM NBT, 0.025 % (v/v) Triton X-100, 2.4 µM riboflavin (as the source of superoxide radicals) and the protein extract. After adding riboflavin, the reaction mixtures were irradiated (300 µmol m^−2^ s^−1^, provided by three 23 W Osram DULUX PRO compact fluorescent lamps) for 10 min at 25 °C, and the absorbance at 560 nm was measured using a non-irradiated reaction mixture as a blank. One SOD unit was defined as the amount of enzyme that causes 50 % inhibition of NBT photoreduction under assay conditions.

##### Catalase

Catalase activity was determined as described by Aebi (1984), following the decrease in absorbance at 240 nm which accompanied the consumption of H_2_O_2_ (Δ*ɛ* = 39.4 mM^−1^ cm^−1^) after adding the protein extracts to a 10 mM H_2_O_2_ solution in 50 mM Tris–HCl (pH 7.0). One CAT unit was defined as the amount of enzyme that will decompose 1 µmol of H_2_O_2_ per minute at 25 °C.

##### Glutathione reductase

Glutathione reductase activity was determined according to Connell and Mullet (1986), following the oxidation of NADPH, the cofactor in the GR-catalysed reduction of oxidized glutathione (GSSG). In a 1-mL final volume, the reaction mixtures contained 100 mM Hepes, pH 7.5, 1 mM EDTA, 3 mM MgCl_2_, 0.5 mM GSSG and the protein extracts. Reactions were initiated by adding NADPH to a final concentration of 0.2 mM. Samples were incubated at 25 °C, and the decrease in absorbance at 340 nm (Δ*ɛ* = 6.22 mM^−1^ cm^−1^) was measured after 25 min. Control reactions with no protein extract were incubated in parallel to correct for non-enzymatic NADPH oxidation. One GR unit was defined as the amount of enzyme that will oxidize 1 µmol of NADPH per minute at 25 °C.

### Statistical analysis

All quantitative data obtained on plant biochemical markers and soil and climatic parameters were analysed statistically using the program STATGRAPHICS Centurion v.16 (Statistical Graphics Corp.). Analysis of variance (one-way ANOVA) was applied with a minimum confidence interval of 95 %, considering the ‘sampling date’ as the grouping factor. When the null hypothesis was rejected, post hoc comparisons were performed with Fisher's least significant difference (LSD) test to discriminate among the means for each dependent variable. Prior to the analysis, ANOVA requirements were checked by normality plots and by testing the homogeneity of variance of the residual means. Biochemical markers in plant material were analysed separately for each species.

Additionally, the XLSTAT v.2013.4.03 (AddinSoft SARL) program was used to perform the PCA, to project the variables in biplots whose components (PC1, PC2) accounted for most of the observed variability. Significant covariances between the studied parameters were defined by correlation matrices using the Pearson correlation coefficient (*r*) (*P* < 0.05) as an index of similarity. The squared cosine of variables was used to define those with the largest contribution to the PC1 axis of inertia.

## Results

### Seasonal changes in soil and climatic conditions at the study site

The Mediterranean climate is characterized by an uneven distribution of precipitation, with a drastic reduction in summer and an increase in autumn and spring (Rivas-Martínez and Rivas-Saenz 1996–2009). However, the 2 years of this study were quite different in meteorological terms (Table 1). The first sampling in summer 2009 was preceded by a strong drought, with practically no accumulated rainfall during the previous 2 months, whereas in the summer of 2010 rainfall was significantly higher; however, average temperatures were similar. Moreover, the dry summer of 2009 was followed by an extremely wet autumn, in contrast to the dry, but significantly cooler, autumn of year 2. It is also characteristic of the Mediterranean climate that, in summer, ETP is considerably greater than precipitation and water deficit is pronounced. In our case, water deficit was greater in 2009 than in 2010 due to the differences in precipitation, although ETP was similar in the two summers. During this season, the water table decreased and the soil surface became dry, resulting in very low soil moisture in both years (Table 1).

**Table 1.** Seasonal changes in soil and climatic variables in the sampling zone. EC_1:1_, electrical conductivity (1 : 1 w/v); *θ*, soil volumetric humidity; AvT, average temperature; P, precipitation; ETP, evapotranspiration; WD, water deficit. EC_1:1_ and *θ* values shown are the means (with standard deviations) of three random samples collected in the experimental zone (*n* = 3). Temperature values correspond to the means (±SD) of weekly data during the 2 months prior to the sampling day, in each season (*n* = 8). ‘Accumulated’ values are the sum of weekly data during the 2 months prior to the sampling day (*n* = 8), in each season. ^1^Numbers followed by the same letter within a column are not significantly different (*P* > 0.05; ANOVA followed by LSD).

Sampling	Mean ± SD^1^			Accumulated (2 months)		
	EC_1:1_ (dS m^−1^)	*θ* (cm^3^ cm^−3^)	AvT (°C)	*P* (mm)	ETP (mm)	WD (mm)
Summer 2009	2.81 ± 0.61a	0.004 ± 0.001a	21.88 ± 2.39ab	8.4	286.77	−278.37
Autumn 2009	0.52 ± 0.16bc	0.140 ± 0.026c	19.29 ± 1.79a	324.8	143.46	181.34
Spring 2010	0.21 ± 0.03c	0.176 ± 0.020d	12.07 ± 2.90d	113	136.95	−23.95
Summer 2010	2.66 ± 0.55a	0.019 ± 0.007a	22.41 ± 2.58b	55	285.90	−230.90
Autumn 2010	1.33 ± 0.28b	0.110 ± 0.010b	14.67 ± 2.96c	75.8	119.88	−44.08

The soil in the study area had a sandy texture (96.2 % sand, 0.6 % silt, 3.2 % clay) and was alkaline, with a mean pH value of 8.9, which did not vary significantly among seasons, nor did other soil properties such as oxidizable organic carbon or water-holding capacity (Gil *et al.* 2011). In a previous study, we found no significant differences in pH across the site, or even when comparing several salt marshes in the ‘La Albufera’ Natural Park (Lidón *et al.* 2009; and A. Lidón, unpublished results).

Strong seasonal variation in soil EC_1:1_ was detected in the salt marsh, which correlated with the meteorology of the study period (Table 1). In summer, high ETP causes an upward movement of water with dissolved salts that accumulate, in part precipitated, in the soil upper layers, explaining the high EC_1:1_ measured in 2009 and 2010. Autumn 2009 was very rainy and this led to surface salts leaching into the water table to give lower EC_1:1_ values; because autumn 2010 was much drier than autumn 2009, autumnal salinity on the soil surface in 2009 was more than double than in 2010. Spring was the season with the lowest soil EC_1:1_, relatively low average temperatures, ETP and water deficit and relatively high precipitation and soil moisture (Table 1).

The wide seasonal changes in soil EC_1:1_ were mostly due to strong and parallel fluctuations of Cl^−^ and Na^+^ concentrations, by far the most abundant ions accumulated by seawater aerosols on the ground of coastal areas (Table 2). Very low soil levels with slight, non-significant seasonal variation were detected for K^+^ (Table 2), which can be explained by the very low cation exchange capacity of sandy soils. Ca^2+^ and Mg^2+^ levels were regulated by the equilibrium solubility of the corresponding carbonates. Calcium is mostly precipitated as CaCO_3_ while MgCO_3_ is soluble and remains in the soil solution; this means that Mg^2+^ concentrations, as occurs with Na^+^ and Cl^−^, vary with seasonal cycles of rise and fall of soil moisture.

**Table 2.** Seasonal changes in soil ion contents in the sampling zone. Ion measurements were performed in 1 : 1 (w/v) aqueous soil extracts; all values refer to soil DW. Cl^−^, chloride; Na^+^, sodium; K^+^, potassium; Ca^2+^, calcium; Mg^2+^, magnesium. The values shown are the means (with standard deviations) of three random samples collected in the experimental zone (*n* = 3). ^1^Numbers followed by the same letter within a column are not significantly different (*P* > 0.05; ANOVA followed by LSD).

Sampling	Mean ± SD^1^				
	Cl^−^ (mmol kg^−1^)	Na^+^ (mmol kg^−1^)	K^+^ (mmol kg^−1^)	Ca^2+^ (mmol kg^−1^)	Mg^2+^ (mmol kg^−1^)
Summer 2009	30.47 ± 7.24d	26.71 ± 6.05c	0.57 ± 0.42a	3.45 ± 0.51a	2.03 ± 0.81c
Autumn 2009	4.20 ± 1.82b	3.26 ± 0.91a	0.50 ± 0.15a	3.28 ± 0.83a	0.67 ± 0.24a
Spring 2010	0.94 ± 0.28a	3.23 ± 0.67a	0.52 ± 0.15a	5.89 ± 0.94b	0.70 ± 0.03a
Summer 2010	22.84 ± 9.30d	20.00 ± 10.42c	0.70 ± 0.37a	3.65 ± 1.69a	1.52 ± 0.44bc
Autumn 2010	9.20 ± 4.32c	8.32 ± 3.06b	0.55 ± 0.10a	3.88 ± 0.90a	1.08 ± 0.33ab

### DW ratios in collected plant material

**Figure 1.**
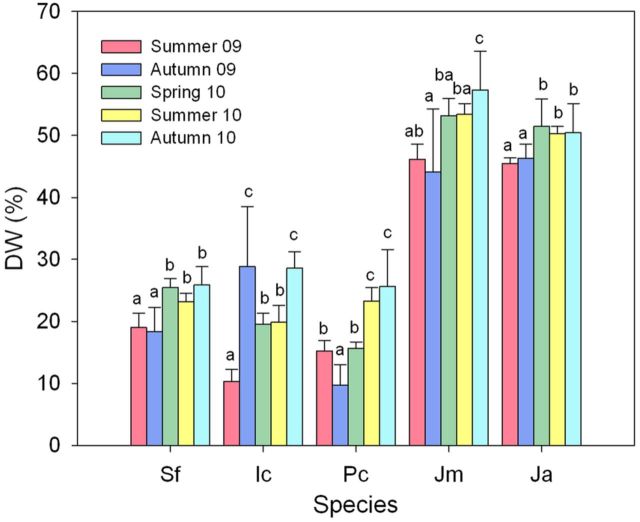
Seasonal variation of DW percentages (DW %) in samples of the five analysed halophytes. Plant material from the aerial parts was collected in the field, in five successive samplings during the years 2009–10, as indicated, and the DW/fresh weight ratio was calculated for each sample. Scale bars represent the means and standard deviations of five independent samples per season and per species; plant material was collected from the same individual plants in all samplings. Different lower case letters indicate significant differences among samplings for each taxon (*α* = 0.05). Plant species: Sf, *Sarcocornia fruticosa*; Ic, *Inula crithmoides*; Pc, *Plantago crassifolia*; Jm, *Juncus maritimus*; Ja, *J. acutus*.

We referred all the measurements of osmolyte and ion concentrations in the selected species to DW of the samples collected from the plant aerial parts. To check whether environmental-dependent variations in the water content of the samples could mask seasonal changes in the levels of the analysed biochemical markers, the DW/fresh weight ratio was determined for all the analysed samples (Fig. 1). The dicotyledonous taxa, *S. fruticosa*, *I. crithmoides* and *P. crassifolia*, were all succulent, as reflected in their relatively low percentage of dry matter, ∼20 %, on average, when compared with the two rushes, *J. maritimus* and *J. acutus*, which showed average DW ratios of ∼50 %. In general, the DW percentages for each species did not vary much between different samplings, especially in *S. fruticosa*, *J. maritimus* and *J. acutus*. These changes were more pronounced in *I. crithmoides* and *P. crassifolia*, but apparently did not correspond to the degree of environmental stress to which the plants had been subjected at the time of sampling (Fig. 1).

### Ion contents

**Figure 2.**
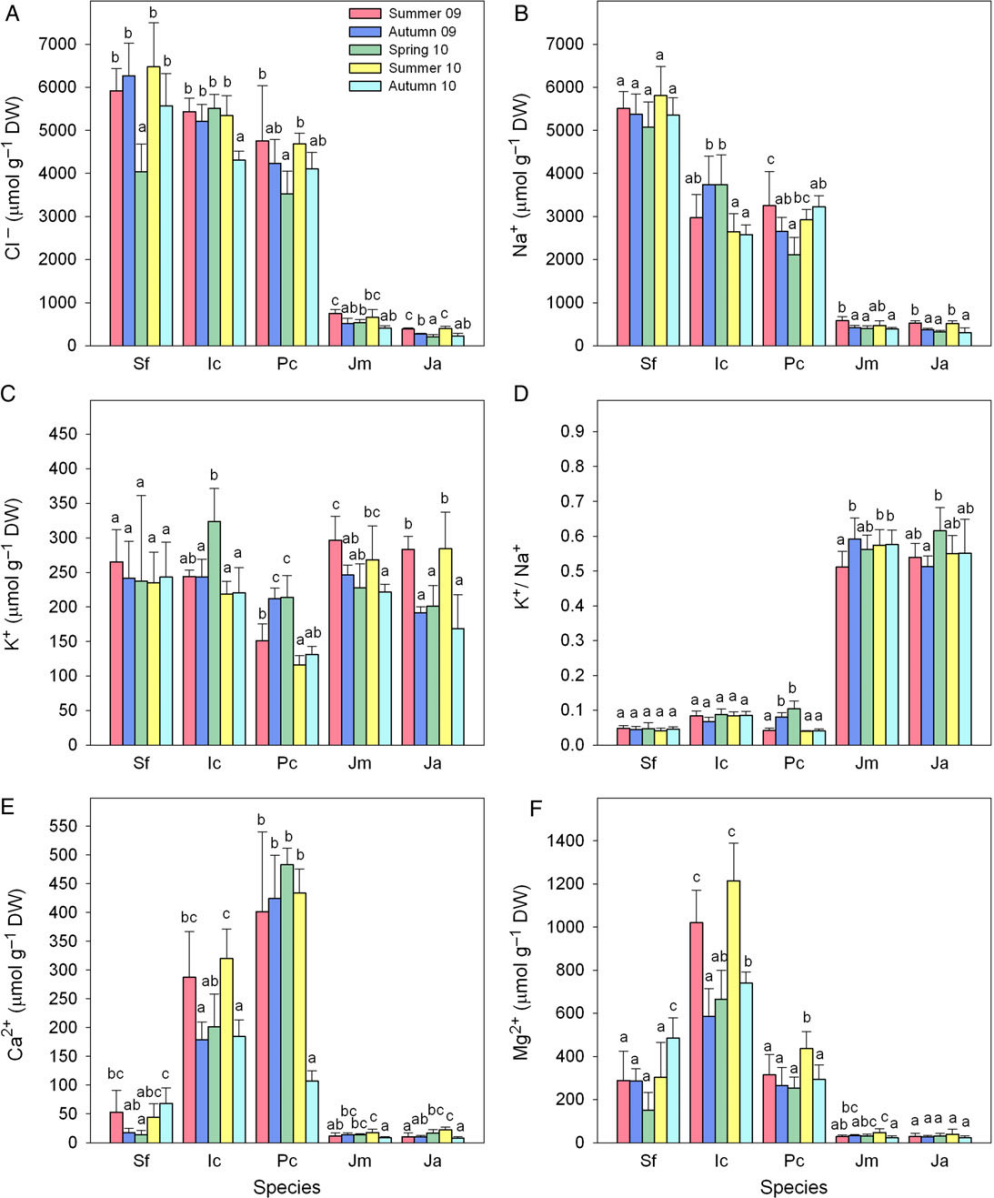
Seasonal variation in ion contents (µmol g^−1^ DW, means ± SD, *n* = 5) in the five selected halophytes. Chloride (A), sodium (B), potassium (C), potassium/sodium ratio (D), calcium (E) and magnesium (F) were determined in the same samples described in Fig. 1. In each panel, different lower case letters indicate significant differences among samplings for each taxon (*α* = 0.05). Plant species abbreviations as in Fig. 1.

Measurements of the Cl^−^ and Na^+^ contents in plants revealed clear differences between dicotyledonous and monocotyledonous taxa, with ∼ten-fold higher concentrations in the former than in the latter (Fig. 2A and B). Specifically, the average Cl^−^ concentrations were ∼5.6, 5.2 and 4.3 mmol g^−1^ DW in *S. fruticosa*, *I. crithmoides* and *P. crassifolia*, respectively, *vs.* 0.58 mmol g^−1^ DW in *J. maritimus* and 0.30 mmol g^−1^ DW in *J. acutus*. Regarding Na^+^ concentrations, the corresponding values were 5.4, 3.1 and 2.8 mmol g^−1^ DW in the dicots, and 0.45 and 0.42 mmol g^−1^ DW in the two *Juncus* species. Seasonal variations in Cl^−^ and Na^+^ concentrations were generally minor and not significant for *S. fruticosa* and *I. crithmoides*. In contrast, seasonal differences in *P. crassifolia*, *J. maritimus* and *J. acutus*, although not very pronounced in absolute terms, were statistically significant and correlated qualitatively with variations in EC_1:1_ and in the levels of Cl^−^ and Na^+^ in soil; that is, with the intensity of salt stress in the field (Tables 1 and 2).

Potassium concentration, however, was similar in all the species investigated, with average values between 165 µmol g^−1^ DW in *P. crassifolia* and 296 µmol g^−1^ DW in *J. maritimus* (Fig. 2C). Once again, no seasonal variations were observed in *S. fruticosa* and *I. crithmoides*. In the remaining taxa, statistically significant changes in K^+^ concentrations were detected, although the concentration of this cation in soil did not change (Table 2). In *P. crassifolia*, the lowest K^+^ levels were measured in the summers of 2009 and 2010, when the highest Cl^−^ and Na^+^ concentrations were detected; conversely, the highest K^+^ values were observed in spring 2010, and corresponded to the lowest contents of toxic ions in the plants. Interestingly, monocotyledonous halophytes showed an opposite behaviour: the seasonal pattern of K^+^ concentration in both *Juncus* species paralleled those of Na^+^ and Cl^−^, with the highest levels recorded in summer and the lowest in spring (Fig. 2C).

Given the Na^+^ and K^+^ accumulation patterns in the different taxa, the K^+^/Na^+^ ratio was relatively high in the two rush species, ∼0.6 on average, while it remained <0.1 in the dicots, *S. fruticosa*, *I. crithmoides* and *P. crassifolia*. Seasonal variations in the K^+^/Na^+^ ratios were not significant in *S. fruticosa* and *I. crithmoides* nor in the other three taxa in most cases, although the mean K^+^/Na^+^ values in *P. crassifolia* showed a rough *negative* correlation with changes in soil salinity (EC_1:1_ values), as they were highest in spring and lowest in summer (Fig. 2D).

The accumulation patterns of the divalent cations, Ca^2+^ and Mg^2+^, in dicotyledonous plants were variable, depending on species, although the average values were higher in all cases than those measured in *Juncus*, similar to the observations for Na^+^ and Cl^−^ (Fig. 2E and F). The lowest Ca^2+^ concentrations in the dicots were measured in *S. fruticosa* (between 14 and 68 µmol g^−1^ DW) and the highest in *P. crassifolia* (400–480 µmol g^−1^ DW), while *I. crithmoides* showed intermediate values (180–320 µmol g^−1^ DW) (Fig. 2E). Significant seasonal variations were observed in calcium concentrations in *S. fruticosa* and *I. crithmoides*, with fluctuations that more or less followed changes in soil salinity in the field, especially in *I. crithmoides*; i.e. with higher values in both summers than in spring. In contrast, these significant seasonal variations in Ca^2+^concentrations were not observed in *P. crassifolia* (Fig. 2E). The highest Mg^2+^ levels were detected in *I. crithmoides* (600–1200 µmol g^−1^ DW), with a significantly higher accumulation in summer than in spring or autumn. *Sarcocornia fruticosa* and *P. crassifolia* showed lower levels of Mg^2+^, and seasonal variations were generally not statistically significant (Fig. 2F). Very low divalent cation concentrations were measured in both *Juncus* species (13 µmol g^−1^ DW for calcium and ∼30 µmol g^−1^ DW for magnesium, on average), with no significant variations in the different samplings (Fig. 2E and F).

### Osmolyte contents

**Figure 3.**
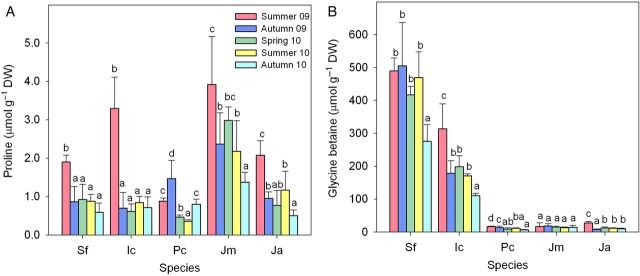
Seasonal variation in proline (A) and glycine betaine (B) contents (µmol g^−1^ DW, means ± SD, *n* = 5) in the five analysed taxa. In each panel, different lower case letters indicate significant differences among samplings for each taxon (*α* = 0.05). Plant samples and species abbreviations as in Fig. 1.

Proline (Pro) is probably the commonest osmolyte in plant species, but it does not seem to be the main compatible solute in any of the five taxa selected for this study. The absolute levels of accumulated Pro were very low, <4 µmol g^−1^ DW in all cases (Fig. 3A), and significantly higher in the most stressful sampling period, summer 2009, except for *P. crassifolia*. The most salt-tolerant taxa, *S. fruticosa* and *I. crithmoides*, were typical GB accumulators (Fig. 3B). Glycine betaine levels reached ∼500 µmol g^−1^ DW in the former species, and up to 300 µmol g^−1^ DW in the latter species, with generally non-significant seasonal fluctuations, not related in any case to the degree of water or salt stress in the sampling area, except for *I. crithmoides* in summer 2009. Much lower GB levels were detected in the remaining species, below ∼20 µmol g^−1^ DW and with no appreciable seasonal changes (Fig. 3B).

### Oxidative stress assessment and antioxidant systems

Unexpectedly, in general only minor seasonal fluctuations in MDA concentrations were detected in all the investigated taxa; in any case, these differences were not statistically significant and/or did not correlate with external stress intensity. Similarly, a lack of significant seasonal variations correlated with the degree of environmental stress was observed when the levels of total phenolic compounds and flavonoids (as examples of non-enzymatic antioxidants) or the specific activities of enzymatic antioxidant systems, SOD, CAT and GR, were determined in the plant samples **[see Supporting information]**.

### Principal component analysis

**Figure 4.**
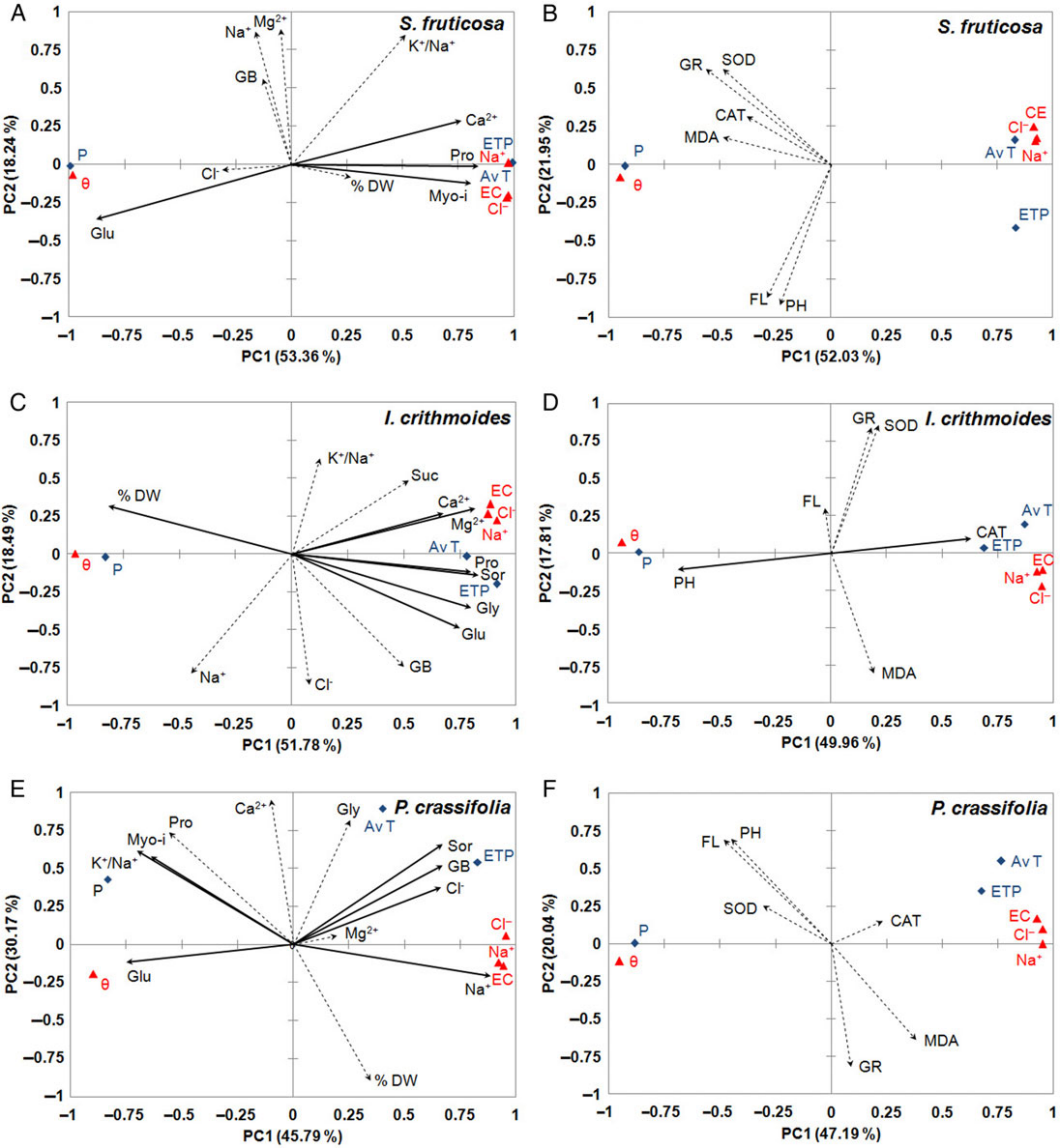
Site score plot of the studied variables on the two principal components (PC1, PC2) for dicotyledonous halophytes *S. fruticosa* (A, B), *I. crithmoides* (C, D) and *P. crassifolia* (E, F). Principal component analyses included, as the analysed variables, those related to osmotic adjustment (osmolytes and ions) (A, C, E) or those related to oxidative stress and enzymatic and non-enzymatic antioxidant systems (B, D, F). Plotted points belong to the soil (red triangles) and climate (blue diamonds) variables, and arrows to those of plants; continuous lines indicate that, for the corresponding variable, the largest inertia determined by the squared cosines belongs to PC1, and dashed lines refer to those variables which do not comply with this. Symbols—DW %, percentage of DW; Pro, proline; GB, glycine betaine; carbohydrates: Fru (fructose), Glu (glucose), Gly (glycerol), Myo-i (*myo*-inositol), Sor (sorbitol) and Suc (sucrose) (only those carbohydrates that correlated with environmental stress variables are included in the graphs). Soil and climatic variables: AvT, average temperature; EC, electrical conductivity in 1 : 1 soil extracts, EC_1:1_; ETP, evapotranspiration; P, precipitation. Ions: Cl^−^, chloride; Na^+^, sodium; K^+^, potassium; Ca^2+^, calcium; Mg^2+^, magnesium; K^+^/Na^+^, potassium/sodium ratio.

**Figure 5.**
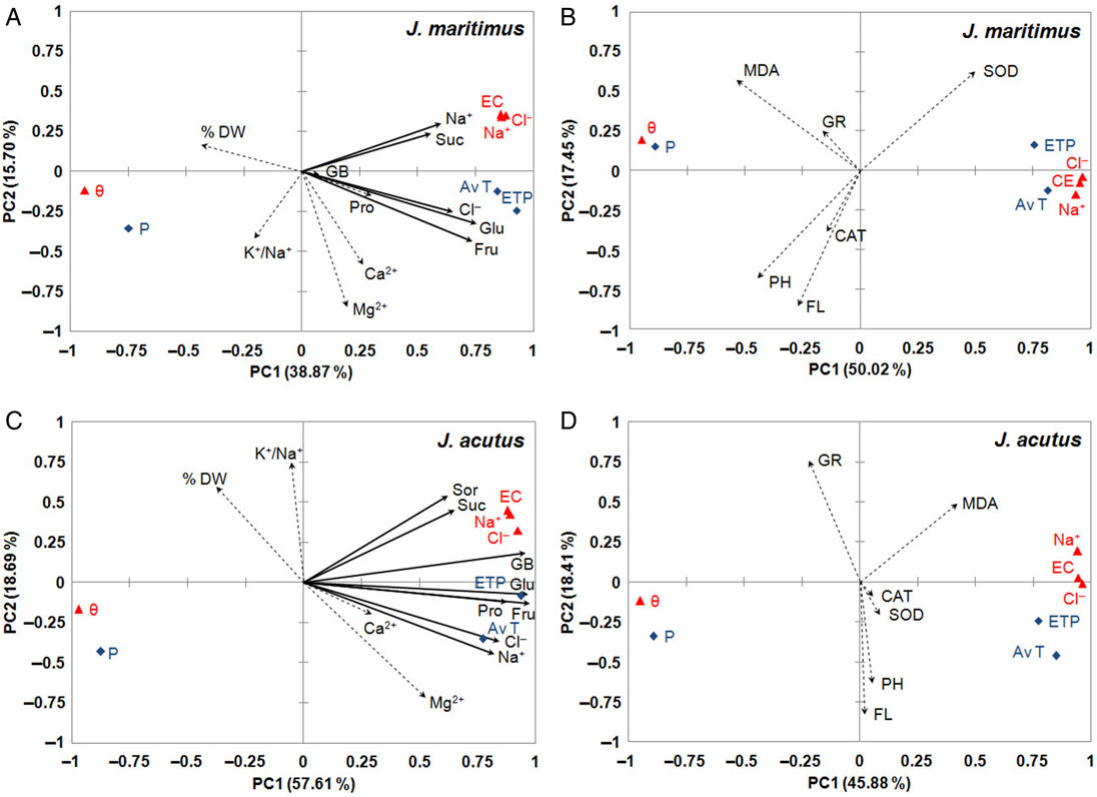
Site score plot of the studied variables on the two principal components (PC1, PC2) for monocotyledonous halophytes *Juncus maritimus* (A, B) and *J. acutus* (C, D). Principal component analyses included, as the analysed variables, osmolytes and ions (A, C) or those related to oxidative stress and antioxidant systems (B, D). Symbols as in Fig. 4.

Two independent PCAs of the experimental data were carried out separately for each species investigated, to establish whether there were statistically significant correlations between the changes in the levels of biochemical stress markers and the fluctuations in the selected environmental parameters associated with water and salt stress (Figs 4 and 5). The first PCA referred to those markers related to ion homoeostasis and to the maintenance of cellular osmotic balance: water content in plants (estimated as DW percentages), inorganic ions (Cl^−^, Na^+^, Ca^2+^, Mg^2+^ and also K^+^/Na^+^ ratios) and osmolyte (Pro and GB) levels. We also included in this analysis all raw experimental data of soluble carbohydrate (sugars and polyalcohols) contents previously determined by high-performance liquid chromatography in the same plant samples (Gil *et al.* 2011); nonetheless, only those that correlated with soil or climatic parameters, according to the newly performed PCAs, are shown in the graphs. The second PCA included the variables related to oxidative stress and antioxidant defence mechanisms: MDA levels, total phenolic compounds and flavonoid contents, and SOD-, CAT- and GR-specific activities. All the analyses gave Eigenvalues greater than one. The first component, PC1, of the PCAs represented in the abscissa, generally showed a high positive correlation with soil properties and climatic data related to water stress (temperature and ETP) and to salt stress (EC_1:1_ and Na^+^ and Cl^−^ contents in soil). Consequently, the negative axis of the PC1 component correlated with precipitation and soil moisture, parameters that can be associated with a low degree of water and salt stress (Figs 4 and 5).

In *S. fruticosa*, the scree plot of the first PCA (Fig. 4A) shows that components PC1 and PC2 jointly explained ∼72 % of observed variability (53 % for PC1 alone). In this case, the variables with greater squared cosines for PC1 corresponded to Pro, Ca^2+^, glucose and *myo*-inositol. However, the Pearson correlation coefficients were significant (*P* < 0.05) only for Pro, which correlated positively with average temperature (AvT) and ETP (*r* = 0.823 for both) and negatively with precipitation (*r* = −0.823). Variations in DW percentages, Cl^−^, Na^+^ and Mg^2+^ contents, K^+^/Na^+^ ratio and GB levels did not correlate with the selected edaphic and climatic variables (Fig. 4A).

In *I. crithmoides*, both components PC1 and PC2 explained 70 % of observed variability, of which 52 % corresponded to PC1 (Fig. 4C). Divalent cations (Ca^2+^ and Mg^2+^), Pro, glucose, sucrose, glycerol and sorbitol correlated significantly with the soil and climatic variables, according to the Pearson correlation coefficients. For example, Pro correlated positively with ETP (*r* = 0.611), EC_1:1_ (*r* = 0.698) and the Cl^−^ and Na^+^ contents in soil (*r* = 0.676, 0.775, respectively), and negatively with precipitation (*r* = −0.806) and soil moisture (*r* = −0.784). As in *S. fruticosa*, no correlation of the monovalent ions or the GB variables with the stress-related environmental parameters was detected (Fig. 4C).

In *P. crassifolia*, in the biplot, PC1 and PC2 explained 76 % of total variability, with 46 % corresponding to PC1. In this particular species, however, the climatic variables associated with water stress, AvT and ETP (positively), or precipitation (negatively), did not correlate as closely with the PC1 axis as in the other taxa. The previously established correlation of sorbitol—the major osmolyte in this species—with the selected environmental parameters (Gil *et al.* 2011) was confirmed in this analysis, which included all the additional variables. Thus, sorbitol correlated positively with AvT (*r* = 0.807) and ETP (*r* = 0.914), but negatively with soil moisture (*r* = −0.759). In addition, significant correlations were detected for some of the newly analysed variables, including GB which, like sorbitol, correlated positively with AvT and ETP (*r* = 0.812, 0.816, respectively). The plant Na^+^ and Cl^−^ concentrations also correlated with the PC1 axis in *P. crassifolia*, but Pro did not (Fig. 4E), unlike the observation for the two previous taxa. The significant negative correlation of the K^+^/Na^+^ ratio with axis PC1 should also be noted.

The scree plots corresponding to the second PCA, shown in Fig. 4B (*S. fruticosa*), Fig. 4D (*I. crithmoides*) and Fig. 4F (*P. crassifolia*), indicated that the squared cosines of all the variables associated with oxidative stress and antioxidant defence were greater for components other than PC1; the Pearson coefficients confirmed that there were no correlations between these variables and the stress-related environmental parameters. The only exception seemed to be the CAT-specific activity in *I. crithmoides*, which correlated positively with soil properties such as EC_1:1_ (*r* = 0.719).

In *J. maritimus*, the scree plot revealed that components PC1 and PC2 together explained 55 % of observed variability, with 39 % ascribed to PC1 alone (Fig. 5A). The squared cosines of the variables corresponding to the plant ion contents (Na^+^ and Cl^−^) and sugar contents (sucrose, fructose and glucose) were greater for component PC1, indicating a possible correlation with the soil and climatic parameters associated with salt and water stress. For example, the pair-wise comparisons using the Pearson correlation coefficients indicated that glucose and fructose correlated significantly and positively with AvT (*r* = 0.683, 0.751), ETP (*r* = 0.670, 0.713) and EC_1:1_ (*r* = 0.595, 0.568), but negatively with soil moisture (*r* = −0.591, −0.611). These data also confirmed previous PCAs, including only the soluble carbohydrate contents in this species and the environmental variables associated with soil salinity and water stress (Gil *et al.* 2011).

In *Juncus acutus*, component PC1 explained 58 % of total variability (Fig. 5B) and the correlation patterns of the plant variables with this component were similar to those observed for *J. maritimus*: plant monovalent ions correlated with the stress-related edaphic and climatic parameters. In this case, however, Pro and GB also correlated with axis PC1, positively with ETP (*r* = 0.859, 0.956), the soil contents of Cl^−^ (*r* = 0.972, 0.773) and Na^+^ (*r* = 0.913, 0.767), but negatively with soil moisture (*r* = −0.923, −0901).

As for dicotyledonous halophytes, the PCAs performed on the experimental data obtained for *J. maritimus* (Fig. 5B) and *J. acutus* (Fig. 5D) did not reveal any significant correlation of the environmental parameters associated with water and salt stress with the oxidative stress markers and antioxidant systems.

## Discussion

Here we describe the analysis of the responses to seasonal changes in the environmental conditions of five selected halophytes, growing in their natural habitat in a Mediterranean littoral salt marsh during a nearly 2-year period. To our knowledge, this is the first systematic study correlating changes in the plants' contents of biochemical markers characteristic of different conserved responses to abiotic stress with variations in a number of soil and climatic parameters associated with environmental stress, and including several mono- and dicotyledonous halophytes. It is true that there are a few previous field studies using a similar strategy (e.g. Murakeözy *et al.* 2002, 2003; Walker *et al.* 2008; Mouri *et al.* 2012; Boscaiu *et al.* 2013), but with a much more limited scope: they usually involved a single halophytic species and shorter study periods, with only a few environmental and plant variables being considered, and/or no statistical analyses of correlations between those variables presented.

While field studies on environmentally induced changes in stress markers are very scarce, there are many reports describing quantitative analyses of ion and osmolyte contents in halophytes growing in the same habitat, thus allowing a comparison of the patterns obtained under the same environmental conditions in different species (e.g. Albert and Popp 1977, 1978; Gorham *et al.* 1980; Briens and Larher 1982; Popp and Polania 1989; Tipirdamaz *et al.* 2006). These and other studies have revealed that salt tolerance in monocotyledonous halophytes, in general, is mostly based on the exclusion of Na^+^ and Cl^−^ from the aerial parts of the plant, the maintenance of higher cellular K^+^/Na^+^ ratios than in dicots and the preferential accumulation of sugars and polyalcohols as compatible solutes for osmotic adjustment (Albert and Popp 1977, 1978; Gorham *et al.* 1980; Briens and Larher 1982; Rozema 1991; Gil *et al.* 2013). Conversely, in dicotyledonous salt-tolerant plants, tolerance mechanisms appear to be primarily based on the transport of Na^+^ and Cl^−^ ions to plant aerial parts, which significantly lowers K^+^/Na^+^ ratios; toxic ions are efficiently transported and stored at high concentrations in the vacuole, and osmotic balance is maintained by accumulation in the cytoplasm of different types of osmolytes, although soluble carbohydrates seem less important for salt tolerance than in monocots (Albert and Popp 1978; Gorham *et al.* 1980; Briens and Larher 1982; Tipirdamaz *et al.* 2006; Gil *et al.* 2013).

The results of the present work generally agree with the aforementioned published data. The five selected halophytes share the same habitat, yet Na^+^ and Cl^−^ concentrations in the two monocotyledonous taxa, *J. maritimus* and *J. acutus*, were much lower than those determined in the dicotyledonous species included in the study—*S. fruticosa*, *I. crithmoides* and *P. crassifolia*—while average K^+^/Na^+^ ratios were ∼6-fold higher in the monocot species. We also observed a relatively higher accumulation of divalent cations, Ca^2+^ and Mg^2+^, in halophytic dicots, which, as far as we know, has not been previously reported. Regarding the compatible solutes used by these species for osmotic balance under stressful conditions, our previous data (Gil *et al.* 2011), which have been confirmed in the present study, indicated that soluble sugars (sucrose, fructose and glucose) are the major osmolytes in the monocots, *J. acutus* and *J. maritimus*. On the other hand, very high GB contents were measured in *S. fruticosa* and *I. crithmoides*, supporting the notion that this compound is the main functional osmolyte in these two species. The presence of GB in *I. crithmoides* has been previously described (Adrian-Romero *et al.* 1998), and there are several reports showing that high levels of this compound accumulate in different *Salicornia* (a genus closely related taxonomically to *Sarcocornia*) species (Moghaieb *et al.* 2004; Tipirdamaz *et al.* 2006; Katschnig *et al.* 2013). However, this is the first systematic study on GB accumulation in relation to abiotic stress in *S. fruticosa* and *I. crithmoides*, except for a preliminary report by our own group (Boscaiu *et al.* 2011*b*). The main physiological osmolyte in *Plantago crassifolia* is sorbitol (Gil *et al.* 2011), as has been shown for all the species of the genus *Plantago* studied to date (Flowers *et al.* 2010).

Sodium accumulation is usually accompanied by a drop in the endogenous levels of K^+^, as Na^+^ competes with K^+^ uptake, particularly when its concentration in the soil solution is significantly higher than that of the nutrient (Niu *et al.* 1995; Rodríguez-Navarro 2000), which is the case in our experimental zone. Yet only *P. crassifolia* seemed to follow this general pattern: an increase in Na^+^ concentration in the periods with higher soil salinity corresponded to a decrease in K^+^, in such a way that the levels of the latter in the plants were higher in spring than in summer. The behaviour of the two species of *Juncus*, in relation to seasonal variations in K^+^ was exactly the opposite: changes in the levels of this cation paralleled those of Na^+^ and Cl^−^, that is, high Na^+^ concentrations in the soil not only did not inhibit but actually stimulated K^+^ accumulation in the plants. It would be interesting to establish how the complex regulatory mechanisms of Na^+^ and K^+^ uptake and distribution in plants, mediated by several transport systems (Rodríguez-Navarro and Rubio 2006; Munns and Tester 2008; Hauser and Horie 2010), operate in these monocotyledonous species. Finally, the most salt-tolerant dicot taxa, *S. fruticosa* and *I. crithmoides*, somewhat surprisingly, did not show significant variations in the levels of Cl^−^, Na^+^ and K^+^ when comparing the five samplings of plant material, despite the dramatic changes recorded in soil EC_1:1_, Cl^−^ and Na^+^ (K^+^ concentrations in soil remained virtually constant). Moreover, the contents of the major osmolyte in these species, GB, also did not change significantly throughout the study period. These data can be interpreted as showing that *S. fruticosa* and *I. crithmoides* possess constitutive mechanisms to maintain osmotic balance under conditions of cellular dehydration, based on the accumulation of solutes—both inorganic ions and compatible osmolytes—at high and more or less constant levels, relatively independent of external conditions. This hypothesis would partly explain the induction of relatively small changes in GB levels, in response to artificial salt stress treatments, in other highly salt-tolerant GB-accumulating species, such as *Suaeda fruticosa* (Khan *et al.* 2000), *Salicornia dolichostachya* (Katschnig *et al.* 2013) or *Spartina alterniflora* (Cavalieri 1983). Our results do not exclude the possibility that the plants may respond to changes in environmental conditions by redistribution of solutes among different subcellular compartments, which in any case should be less costly in terms of energy consumption than uptake and transport of external ions and *de novo* synthesis of osmolytes. There is indeed evidence for salt stress-induced changes in the intracellular localization of compatible solutes in *Limonium latifolium* (Gagneul *et al.* 2007), but data on these putative mechanisms are still scarce.

As regards changes in divalent cations, their patterns were quite variable in different species. Only in *I. crithmoides* was a significant correlation with soil salinity detected, suggesting that the accumulation of Ca^2+^ and Mg^2+^ in this species may contribute to its physiological salt tolerance mechanisms. The fact that Ca^2+^ counteracts some of sodium's harmful effects on plants is well-documented (Rengel 1992; Marschner 1995; Bressan *et al.* 1998; Gul and Khan 2006), and we have reported some indirect evidence indicating that Mg^2+^may play a similar role (Boscaiu *et al.* 2011*a*; Grigore *et al.* 2012). This possibility makes sense, considering that the inhibition by Na^+^ of some enzymatic activities is due to displacement of Mg^2+^, used as cofactor by such enzymes, from their active centre (e.g. Albert *et al.* 2000). Thus, an increased concentration of intracellular Mg^2+^ (without reaching toxic levels) may partially counteract the inhibitory effect of Na^+^ and confer some degree of halotolerance.

We would also like to highlight the significant correlation of Pro levels in the plants with the variables associated with environmental stress, in all investigated taxa except *Plantago crassifolia*, suggesting a functional role of this osmolyte in the stress tolerance mechanisms of the remaining four species. Since Pro absolute contents were in all cases extremely low and could not have any substantial effect on osmotic adjustment, even if restricted to the cytoplasm, its contribution to tolerance must be based on its function(s) as a low-molecular-weight chaperone and/or a ROS scavenger (Ashraf and Foolad 2007; Szabados and Savoure 2010; Grigore *et al.* 2011).

The results of the present study that are most difficult to explain, considering previously published data, are those suggesting that halophytes are not subjected to oxidative stress in their natural habitat. Despite large fluctuations in soil salinity and soil water content, in general no significant seasonal changes were detected in the plants' levels of MDA and, in any case, no correlation with environmental variables associated with salt or water stress could be established. MDA, a membrane lipid peroxidation product, is considered to be an excellent general marker of oxidative stress (del Rio *et al.* 2005) and is routinely used to assess the degree of oxidative damage induced in plants by different stress treatments (e.g. Demiral and Türkan 2004; Li 2008; Aghaleh *et al.* 2009; Li *et al.* 2010). In the absence of oxidative stress, it is logical that we failed to detect any significant correlations of levels of antioxidant compounds (total phenolics and flavonoids) or specific activities of antioxidant enzymes in the selected halophytes, with environmental stress factors.

Our results, therefore, apparently contradict overwhelming evidence indicating that salt and water stress treatments cause ROS generation and, consequently, oxidative stress in plants, to which they respond with the activation of enzymatic and non-enzymatic antioxidant systems (Apel and Hirt 2004; Fedoroff 2006; Ashraf 2009; Miller *et al.* 2010; and references therein). Most of these studies have been carried out in crop species, but a similar behaviour has been reported in halophytes (e.g. Parida *et al.* 2004; Ben Amor *et al.* 2006; Sekmen *et al.* 2007; Li 2008; Aghaleh *et al.* 2011; Ozgur *et al.* 2013): in controlled treatments, MDA levels generally increase several fold as a response to increasing external salt concentrations, although its absolute values vary widely in different halophytic species, thus precluding direct quantitative comparisons.

Malondialdehyde and antioxidants have not been determined before in any of the five taxa studied herein, but it seems extremely unlikely that they should behave differently to other plant species. Since most of, if not all, the studies on this specific topic have been conducted under artificial laboratory or greenhouse conditions, and not in natural plant habitats, an explanation for our negative results could be sought in the essential differences between these two types of experimental setups. Firstly, in our study we used several years-old adult plants (they are all perennial taxa), perfectly adapted to their environment in the salt marsh. However, in the greenhouse setting, usually seedlings or young plants obtained by seed germination, but rarely adult plants, are subjected to shock salt treatments. It is well known that salt tolerance of a given species depends largely on the development stage and, specifically regarding vegetative growth, mature plants tend to be more tolerant than younger ones (Johnson *et al.* 1992; Vicente *et al.* 2004). Moreover, in salt-treated potted plants the root system is constrained in a limited environment of homogeneous salinity, while roots in the field can explore a much larger and more heterogeneous soil volume, where significant local differences in salinity occur. There are also data suggesting that absorption of water and nutrients by plants primarily takes place through those roots present in the least saline zones of the soil (reviewed by Bazihizina *et al.* 2012). In short, plants treated in the greenhouse with saline solutions can actually be subjected to salt stress levels that are well above those affecting the same species growing in the field under apparently similar conditions if, that is, those conditions are estimated from surface measurements of electrical conductivity or ion contents in soil.

Bearing this in mind as a plausible explanation for our results, we propose that, in the selected halophytes and under field conditions, stress responses based on the control of ion uptake and compartmentalization, and on the maintenance of cellular osmotic balance by the accumulation of specific osmolytes are efficient enough to avoid excessive ROS production and to reduce ROS levels once formed—also considering the role of compatible solutes as ROS scavengers. Therefore, the activation of antioxidant systems was not detected in the investigated salt-tolerant species simply because they did not need them as a form of defence against environmental stress in their natural habitat.

## Conclusions

The present study confirms some previously published results, but also provides novel information contributing to our knowledge on the general mechanisms of abiotic stress tolerance in plants. The two analysed monocotyledonous halophytes, *J. maritimus* and *J. acutus*, are able to limit the transport of Na^+^ and Cl^−^ ions (but also of Ca^2+^ and Mg^2+^) to the shoots, maintaining relatively high K^+^/Na^+^ ratios, whereas the dicotyledonous taxa included in the study (*S. fruticosa*, *I. crithmoides* and *P. crassifolia*) accumulate these monovalent toxic ions at high levels in their aerial parts, preferentially sequestered in the vacuoles, and synthesize specific osmolytes for intracellular osmotic adjustment. The most salt-tolerant taxa, *S. fruticosa* and *I. crithmoides*, seem to possess constitutive mechanisms of abiotic stress tolerance based on the presence of relatively high and more or less constant levels of inorganic ions and GB (their major compatible solute), independently of changes in environmental conditions. Proline may contribute to stress tolerance mechanisms in all investigated taxa (except *P. crassifolia*)—being present at very low concentrations, which exclude any osmotic effect—due to its role as a low-molecular-weight chaperone and/or a ROS scavenger. Unexpectedly, the selected halophytes do not seem to be affected by oxidative stress in their natural habitat, and consequently do not need to activate antioxidant systems as a defence against environmental stress. We propose that the aforementioned tolerance mechanisms, based on the control of ion transport and osmolyte biosynthesis, are efficient enough to avoid excessive ROS production and oxidative damage in the plants.

## Sources of Funding

This work was funded by a grant to O.V. from the Spanish Ministry of Science and Innovation (Project CGL2008-00438/BOS), with contribution from the European Regional Development Fund.

## Contributions by the Authors

Analyses in plant material were performed by R.G. (osmolytes), J.L. (ions), S.W. (MDA) and H.S. (enzyme assays). M.B. was responsible for the identification, sampling and processing of plant material and the co-supervision of plant-related work. Soil analyses were performed by I.B. and A.L. Statistical analyses and preparation of figures were carried out by R.G., I.B., A.L. and O.V. was responsible for the general coordination of the project, the supervision of the biochemical work and the preparation of the manuscript (with contributions from M.B., R.G., I.B. and A.L. for the sections referring to botanical, soil and statistical aspects).

## Conflicts of Interest Statement

None declared.

## Supporting Information

The following Supporting Information is available in the online version of this article –

**Table S1.** Seasonal changes in the levels of oxidative stress markers and non-enzymatic antioxidants, and in the specific activity of antioxidant enzymes, determined in field-collected material of the selected halophytes.

Additional Information
