# Supplementary material for: Responses of five Mediterranean halophytes to seasonal changes in environmental conditions
Source: AoB Plants. 2014 Aug 19;6:plu049. doi: 10.1093/aobpla/plu049 (PMC4163002; doi:10.1093/aobpla/plu049)
Supplement: Additional Information [file supp_6_plu049_index.html]

Responses of five Mediterranean halophytes to seasonal changes in environmental conditions — Responses of five Mediterranean halophytes to seasonal changes in environmental conditions — Additional Information 

# Responses of five Mediterranean halophytes to seasonal changes in environmental conditions

## Additional Information

Additional Information

**Files in this Data Supplement:**

- Additional Information - docx file
