# Supplementary material for: Responses of five Mediterranean halophytes to seasonal changes in environmental conditions
Source: AoB Plants. 2014 Aug 19;6:plu049. doi: 10.1093/aobpla/plu049 (PMC4163002; doi:10.1093/aobpla/plu049)
Supplement: Additional Information [file supp_plu049_plu049supp.docx]

**Table**. **Seasonal changes in the levels of oxidative stress markers and non-enzymatic antioxidants, and in the specific activity of antioxidant enzymes**, determined in field-collected material of *Sarcocornia fruticosa* (A), *Inula crithmoides* (B), *Plantago crassifolia* (C)*, Juncus maritimus* (D) and *J. acutus* (E). MDA: malondialdehyde; PH: total phenols; FL: flavonoids; SOD: superoxide dismutase; CAT: catalase; and GR: glutathione reductase. Data correspond to means (with standard deviations) expressed on a dry weight basis of samples from five individual plants (n=5). PH and FL were expressed as mg equivalents of gallic acid (GA) and catechin (C), respectively. Enzyme specific activity was expressed as units per mg protein (U mg^-1^ protein). One SOD unit was defined as the amount of enzyme that causes 50% inhibition of NBT photoreduction under the assay conditions. CAT and GR units were defined as the amount of each enzyme needed for decomposing 1 µmol of H_2_O_2_ and NADPH, respectively, per minute at 25ºC.

**A.** *Sarcocornia fruticosa*

| **Sampling** | **Mean ± SD (n = 5)^1^** | | | | | |
| --- | --- | --- | --- | --- | --- | --- |
|  | MDA (µmol  g^-1^ DW) | PH (mg eq. GA  g^-1^ DW) | FL (mg eq. C  g^-1^ DW) | SOD (U  mg^-1^ protein) | CAT (U  mg^-1^ protein) | GR (U  mg^-1^ protein) |
| Summer 2009 | 0.15 ± 0.01a | 9.42 ± 1.04bc | 2.96 ± 0.86bc | 4.88 ± 5.05a | 5.95 ± 1.22a | 0.04 ± 0.01a |
| Autumn 2009 | 0.23 ± 0.05b | 10.84 ± 1.04c | 3.43 ± 0.55c | 10.50 ± 2.86b | 11.19 ± 1.95b | 0.05 ± 0.004b |
| Spring 2010 | 0.12 ± 0.02a | 18.34 ± 1.74d | 8.92 ± 0.77d | 6.58 ± 2.09ab | 14.53 ± 1.32bc | 0.04 ± 0.003a |
| Summer 2010 | 0.09 ± 0.03a | 8.55 ± 1.27ab | 2.61 ± 1.28ab | 6.44 ± 4.56ab | 13.23 ± 2.38bc | 0.04 ± 0.01a |
| Autumn 2010 | 0.23 ± 0.06b | 7.78 ± 0.32a | 1.77 ± 0.28a | 13.28 ± 6.58b | 15.24 ± 4.87c | 0.07 ± 0.004c |

**B.** *Inula crithmoides*

| **Sampling** | **Mean ± SD (n = 5)^1^** | | | | | |
| --- | --- | --- | --- | --- | --- | --- |
|  | MDA (µmol  g^-1^ DW) | PH (mg eq. GA  g^-1^ DW) | FL (mg eq. C  g^-1^ DW) | SOD (U  mg^-1^ protein) | CAT (U  mg^-1^ protein) | GR (U  mg^-1^ protein) |
| Summer 2009 | 0.27 ± 0.05c | 7.7 ± 1.8b | 3.78 ± 1.55a | 35.15 ± 0.17a | 11.41 ± 2.15ab | 0.08 ± 0.002a |
| Autumn 2009 | 0.2 ± 0.11bc | 7.4 ± 0.13b | 3.31 ± 0.42a | 37.32 ± 4.51a | 7.65 ± 3.55a | 0.1 ± 0.01a |
| Spring 2010 | 0.1 ± 0.08ab | 6.57 ± 1.11b | 3.26 ± 0.46a | 47.95 ± 2.2a | 3.5 ± 1.21a | 0.09 ± 0.01a |
| Summer 2010 | 0.1 ± 0.03a | 6.59 ± 0.91a | 3.29 ± 0.47a | 96.26 ± 7.43b | 18.78 ± 6.78c | 0.16 ± 0.05b |
| Autumn 2010 | 0.19 ± 0.08bc | 6.44 ± 0.9ab | 3.67 ± 0.07a | 49.20 ± 16.07a | 16.24 ± 6.42bc | 0.09 ± 0.02a |

**C.** *Plantago crassifolia*

| **Sampling** | **Mean ± SD (n 0 5)^1^** | | | | | |
| --- | --- | --- | --- | --- | --- | --- |
|  | MDA (µmol  g^-1^ DW) | PH (mg eq. GA  g^-1^ DW) | FL (mg eq. C  g^-1^ DW) | SOD (U  mg^-1^ protein) | CAT (U  mg^-1^ protein) | GR (U  mg^-1^ protein) |
| Summer 2009 | 0.19 ± 0.03b | 5.31 ± 0.39a | 2.16 ± 0.25a | 3.52 ± 1.3a | 5.45 ± 4.94ab | 0.04 ± 0.03b |
| Autumn 2009 | 0.19 ± 0.08b | 8.42 ± 1.96b | 4.92 ± 2.12b | 16.63 ± 9.54a | 17.81 ± 16.31b | 0.04 ± 0.01b |
| Spring 2010 | 0.08 ± 0.02a | 8.52 ± 0.86b | 5.95 ± 0.7b | 3.80 ± 3.67a | 2.3 ± 0.5a | 0.04 ± 0.01b |
| Summer 2010 | 0.06 ± 0.02a | 12.08 ± 1.95c | 8.88 ± 2.29c | 5.87 ± 0.11a | 4.61 ± 0.98ab | 0.02 ± 0.004a |
| Autumn 2010 | 0.22 ± 0.09b | 8.34 ± 1b | 4.62 ± 1.04b | 13.11 ± 14.64a | 7.81 ± 4.03b | 0.07 ± 0.01c |

**D.** *Juncus maritimus*

| **Sampling** | **Mean ± SD (n = 5)^1^** | | | | | |
| --- | --- | --- | --- | --- | --- | --- |
|  | MDA (µmol  g^-1^ DW) | PH (mg eq. GA  g^-1^ DW) | FL (mg eq. C  g^-1^ DW) | SOD (U  mg^-1^ protein) | CAT (U  mg^-1^ protein) | GR (U  mg^-1^ protein) |
| Summer 2009 | 0.12 ± 0.02a | 12.73 ± 0.85a | 4.64 ± 0.65a | 9.41 ± 4.52c | 17.98 ± 3.54a | 0.04 ± 0.02c |
| Autumn 2009 | 0.18 ± 0.02b | 13.25 ± 1.49a | 5.11 ± 0.98ab | 3.41 ± 2.93ab | 24.37 ± 2.31b | 0.04 ± 0.01abc |
| Spring 2010 | 0.17 ± 0.04ab | 12.65 ± 2.42a | 4.65 ± 1.51a | 1.32 ± 1.35a | 26.93 ± 1.84bc | 0.04 ± 0.004bc |
| Summer 2010 | 0.14 ± 0.03ab | 12.57 ± 1.2a | 5.62 ± 1.17ab | 2.47 ± 1.71ab | 34.10 ± 1.81d | 0.03 ± 0.004a |
| Autumn 2010 | 0.15 ± 0.06ab | 13.94 ± 1.91a | 6.15 ± 0.94b | 4.18 ± 0.44bc | 28.69 ± 3c | 0.03 ± 0.002ab |

**E.** *Juncus acutus*

| **Sampling** | **Mean ± SD (n = 5)^1^** | | | | | |
| --- | --- | --- | --- | --- | --- | --- |
|  | MDA (µmol  g^-1^ DW) | PH (mg eq. GA  g^-1^ DW) | FL (mg eq. C  g^-1^ DW) | SOD (U  mg^-1^ protein) | CAT (U  mg^-1^ protein) | GR (U  mg^-1^ protein) |
| Summer 2009 | 0.18 ± 0.02b | 10.98 ± 1.03a | 3.11 ± 0.71a | 22.73 ± 9.19ab | 18.57 ± 7.37a | 0.05 ± 0.01bc |
| Autumn 2009 | 0.12 ± 0.03a | 11.88 ± 0.42a | 4.28 ± 0.39c | 21.30 ± 4.74ab | 17.49 ± 4.29a | 0.04 ± 0.01a |
| Spring 2010 | 0.15 ± 0.03a | 10.80 ± 1.92a | 3.28 ± 0.76ab | 25.76 ± 5.91ab | 16.51 ± 2.21a | 0.06 ± 0.01cd |
| Summer 2010 | 0.15 ± 0.02a | 10.97 ± 0.56a | 4.64 ± 1.46c | 27.71 ± 5.51b | 22.95 ± 2.02ab | 0.04 ± 0.002ab |
| Autumn 2010 | 0.13 ± 0.06a | 10.86 ± 0.51a | 4.04 ± 0.40bc | 18.65 ± 4.33a | 35.83 ± 14.43b | 0.07 ± 0.02d |

^1^ Numbers followed by the same letter within a column are not significantly different (P > 0.05, ANOVA followed by LSD)
